# Comprehensive Iranian guidelines for the diagnosis and management of mitochondrial disorders: an evidence- and consensus-based approach

**DOI:** 10.1186/s13023-025-04127-y

**Published:** 2025-12-19

**Authors:** Setila Dalili, Noushin Rostampour, Seyedeh Tahereh Mousavi, Saeid Sadeghi Joni, Nejat Mahdieh, Afagh Hassanzadeh Rad, Seyede Tahoura Hakemzadeh, Sara Nikpour, Ali Talea, Hossein Moravvej

**Affiliations:** 1 Pediatric Diseases Research Center, Guilan University of Medical Sciences, Rasht, Iran; 2 Metabolic Liver Disease Research Center, Isfahan University of Medical Sciences, Isfahan, Iran; 3 Assistant Professor of Pediatrics Endocrinology, School of Medicine, Bushehr University of Medical Sciences, Bushehr, Iran; 4 Department of Radiology, Razi Hospital, Guilan University of Medical Sciences, Rasht, Iran; 5 Cardiogenetic Research Center, Rajaie Cardiovascular Medical and Research Institute, Tehran, Iran; 6 Pediatric Endocrinologist, Metabolic Disorders Research Center, Molecular-Cellular Endocrinology & Metabolism Research Institute, Tehran University of Medical Sciences, Tehran, Iran; 7 Neonatal Research Center, Shiraz University of Medical Sciences, Shiraz, Iran

**Keywords:** Mitochondrial diseases, Diagnosis, Genetic testing

## Abstract

Mitochondrial disorders are a heterogeneous group of inherited metabolic diseases resulting from dysfunctions in oxidative phosphorylation. These conditions predominantly affect high-energy-demand organs such as the brain, heart, liver, and muscles, leading to diverse clinical manifestations and diagnostic challenges. This article presents the first comprehensive Iranian guideline for the diagnosis and management of mitochondrial diseases, developed through an evidence-based and consensus-driven methodology. We conducted a structured literature review across major biomedical databases from 2000 to 2023 and engaged a multidisciplinary panel of Iranian experts to establish context-specific recommendations. The guideline covers clinical presentations, laboratory biomarkers, neuroimaging features, genetic diagnostics, and treatment approaches including “cocktail therapy” and acute management protocols. It also integrates a mitochondrial disease scoring system to standardize diagnosis and provides detailed insights into safe anesthesia practices for affected individuals. Special attention is given to practical implementation in resource-limited settings. These guidelines aim to enhance diagnostic accuracy, optimize management strategies, and improve the quality of life for patients with mitochondrial disorders across Iran and similar healthcare systems.

## Introduction

Mitochondrial disorders are a diverse group of rare, inherited metabolic diseases caused by defects in mitochondrial functions, including oxidative phosphorylation (via the respiratory chain), mitochondrial membrane dynamics, tRNA processing, and mtDNA replication [1]. These dysfunctions impair cellular energy production and other critical mitochondrial processes, primarily affecting high-energy-demand organs such as the brain, heart, muscles, and liver [2].

**Fig. 1:**
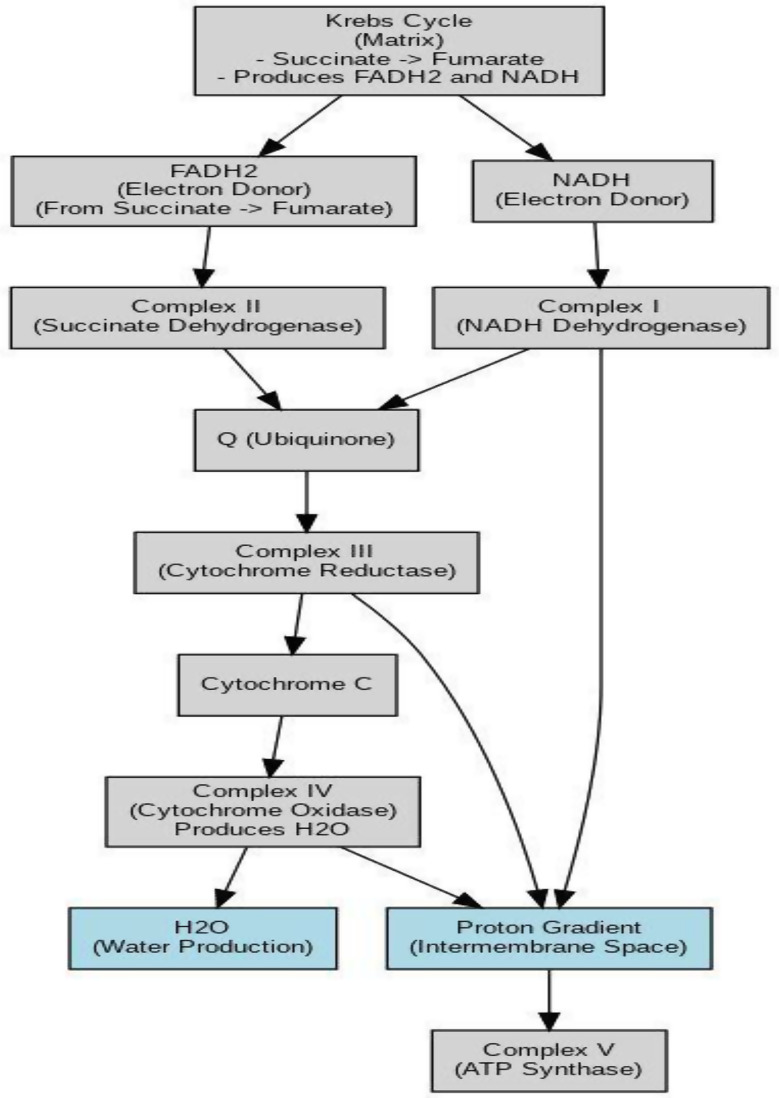
Energy production through krebs cycle and electron transport chain

The mitochondrial respiratory chain consists of five complexes (I–V) embedded in the inner mitochondrial membrane. These complexes are encoded by both nuclear DNA and mitochondrial DNA (mtDNA). Dysfunction in any of these complexes can lead to mitochondrial disease [3]. The mitochondrial respiratory chain is summarized in Fig. 1.

Figure 1 shows that nicotinamide adenine dinucleotide (NADH) and flavin adenine dinucleotide (FADH2) are produced during the Krebs cycle, with FADH2 specifically generated during the conversion of succinate to fumarate. These molecules function as electron carriers. They transfer their electrons to the mitochondrial electron transport chain, where NADH donates its electrons to Complex I. This complex (I) pumps protons into the intermembrane space, while FADH2 donates its electrons to Complex II.

Complex II, unlike Complex I, does not pump protons. Both pathways converge as electrons are transferred to ubiquinone (Q), which delivers them to Complex III. Complex III passes these electrons to cytochrome c, simultaneously pumping additional protons. This process concludes at Complex IV, where oxygen is converted to water (H2O). The accumulation of protons in the intermembrane space creates an electrochemical gradient that drives adenosine triphosphate (ATP) production via ATP synthase (Complex V), converting adenosine diphosphate (ADP) into ATP using the phosphorus [4].

Mitochondrial disorders, which affect approximately 1 in 4,300 individuals globally, arise from defects in this process and primarily manifest in high-energy-demand organs such as the brain, heart, muscles, and liver. Common clinical symptoms include developmental delays, muscle weakness, fatigue, seizures, cardiomyopathy, and endocrine dysfunction, with significant variability between individuals due to genetic and environmental factors [5, 6]. Diagnosing mitochondrial disorders requires a combination of clinical evaluation, biochemical testing, and genetic analysis. Elevated levels of lactate, pyruvate, and alanine in blood or cerebrospinal fluid often suggest mitochondrial dysfunction. As Whole Exome Sequencing (WES) is used to identify variants in nuclear-encoded genes, mitochondrial DNA (mtDNA) sequencing can be a great option for detecting variants in mitochondrial-encoded genes. Functional studies, such as measuring respiratory chain enzyme activity in muscle biopsies or fibroblasts, provide further diagnostic confirmation [7, 8].

Treatment strategies primarily aim to manage symptoms and improve the quality of life for affected individuals. Dietary interventions, such as a high-fat ketogenic diet, may enhance energy metabolism, while cofactor supplementation—including Coenzyme Q10, riboflavin, and L-carnitine—can support mitochondrial function. Emerging therapies, such as gene therapy and mitochondrial replacement techniques, are under active investigation. However, diagnosing and managing mitochondrial disorders in developing countries is particularly challenging due to limited access to advanced diagnostic tools and therapeutic resources, coupled with high costs, lack of insurance coverage for tests, and unavailability of essential supplements. Therefore, developing standardized guidelines tailored to available resources is supportive for improving outcomes in these settings [9].

Regarding the importance of this issue and the shortage of a comprehensive framework for the diagnosis and management of mitochondrial disorders, we aimed to present this review to support healthcare professionals in delivering effective care to affected individuals. This guideline tried to help patients in resource-limited regions utilizing available equipment and instruments.

## Methods

This study was conducted through a comprehensive literature review of articles in English using the keywords “mitochondrial disease,” “mitochondrial dysfunction,” and “respiratory chain disorder” in PubMed, Scopus, Web of Science, Cochrane, and Embase databases from 2000 to 2023. Articles focusing on the clinical presentation, diagnosis, and management of mitochondrial disorders were prioritized.

To ensure a multidisciplinary perspective, cooperation invitations were sent to clinicians and researchers in Iran specializing in inherited metabolic diseases, radiology, and genetic disorders, with a focus on mitochondrial pathologies. These experts held national board certifications in their respective fields. Physicians and scientists who expressed their willingness to contribute were included as authors.

Each participant was assigned a specific section of the article to write based on their expertise. Also, a student was unaware of the mechanisms and she tried to simplify the manuscript to be understandable. Once the sections were completed, the drafts were reviewed and shared among all authors for comments, suggestions, and revisions. Discrepancies were resolved through consensus meetings, during which the authors referred back to the original articles and debated varying viewpoints to achieve an evidence-based and comprehensive set of recommendations.

This collaborative approach ensured that the guidelines were informed by a combination of the latest scientific evidence, clinical expertise, and practical considerations relevant to both resource-rich and resource-limited settings.

### Clinical presentation and systemic involvement in mitochondrial diseases

Mitochondrial diseases exhibit wide-ranging symptoms due to dysfunction in organs with high energy demands. Recognizing these manifestations across systems is critical for timely diagnosis and management.

### Neurological involvement

#### Central nervous system

Metabolic strokes affecting cortex, basal ganglia, midbrain, or brainstem. Seizures, ataxia, migraines, cognitive decline, white matter changes [10].

#### Peripheral nervous system & autonomic dysfunction

Axonal sensorimotor neuropathy, Dysautonomia: orthostatic hypotension, gastrointestinal dysmotility [10].

### Ophthalmologic and Auditory Manifestations

#### Visual symptoms

- Decreased visual acuity not corrected by lenses.
- Photophobia, nyctalopia, visual field deficits due to optic atrophy or retinal disease.
- **Eye Movement Disorders**: Ptosis and progressive ophthalmoplegia [11].
- **Hearing Loss**: Bilateral high-frequency sensorineural hearing loss [11].

### Cardiac involvement

- Cardiomyopathy (often hypertrophic or dilated forms).
- Arrhythmias and conduction abnormalities, sometimes requiring pacemaker insertion.
- Chronic constipation, failure to thrive (12, 13).

#### Gastrointestinal and nutritional problems

- Dysphagia, reflux, delayed gastric emptying.
- Feeding difficulties, pseudo-obstruction, malabsorption.
- Chronic constipation, failure to thrive [12, 13].

#### Endocrine and metabolic dysfunctions

- Pituitary, thyroid, adrenal, and pancreatic dysfunctions.
- Common endocrine complications: hypothyroidism, adrenal insufficiency, diabetes mellitus [14].

#### Sleep disorders

- Poor sleep quality, central or obstructive sleep apnea.
- Can mimic fatigue or daytime somnolence; confirmed with polysomnography [15].

#### Exercise intolerance

- Characterized by reduced VO₂ max and early fatigue.
- Functional testing helps quantify the severity and guides activity plans [16].

#### Clinical implication

- These multisystemic manifestations necessitate a structured, multidisciplinary evaluation to identify treatable features and improve patient outcomes (8).

### Laboratory findings in mitochondrial diseases: metabolic and biochemical indicators

Laboratory tests offer key metabolic clues for mitochondrial dysfunction and help guide diagnosis and management.

### Markers of mitochondrial dysfunction

- Lactate and Pyruvate:
-   Elevated blood and CSF lactate.
-   High lactate-to-pyruvate ratio (> 20) [17].
- Alanine:

- Increased serum and CSF levels [18].
- Diagnostic ratios:
  - Alanine/lysine > 3.
  - Alanine/(phenylalanine + tyrosine) > 4 [8, 19].

### Amino acid profile abnormalities

- Elevated branched-chain amino acids (leucine, isoleucine, valine) [20].
- Elevated proline.
- Generalized aminoaciduria suggesting proximal tubulopathy or mitochondrial dysfunction [21].

### Krebs cycle and organic acid defects

- Raised intermediates: α-ketoglutarate, succinate, fumarate [22].
- Urine organic acid abnormalities:
  - 3-methylglutaconic acid [23].
  - Ethylmalonic acid [24].
  - Methylmalonic acid [24].
  - Dicarboxylic acids (suggest impaired β-oxidation) [18].

### Fatty acid oxidation and biomarkers

- Abnormal **acylcarnitine profiles** (accumulated long- or medium-chain species) [25].
- **GDF-15**: Elevated in mitochondrial myopathies and depletion syndromes [18].

### CSF findings

- Elevated lactate and alanine [23].
- Often correlate with central nervous system (CNS) symptoms such as seizures or ataxia [26].

### Hematologic and endocrine labs

- Anemia, leukopenia, or thrombocytopenia in some patients.
- Elevated creatine kinase (CK) in muscle involvement.
- Abnormal thyroid function, blood glucose, or lipid profiles [9, 27, 28].

Overall, these laboratory findings provide a comprehensive picture of the metabolic disturbances associated with mitochondrial diseases. They are helpful for guiding further diagnostic evaluations and tailoring management strategies for affected individuals.

### MRI findings in mitochondrial disorders

Although advanced neuroimaging and genomic techniques such as MRI/MRS and exome sequencing have considerable diagnostic value, their limited accessibility and high cost often reduce their utility in resource-constrained settings. Therefore, while these modalities remain important, greater emphasis on more readily available tools—including conventional MRI, metabolic screening, and thorough clinical evaluation—is crucial for practical application in low-resource regions. Mitochondrial disorders can manifest in diverse and sometimes unpredictable ways on the brain MRI. These imaging findings reflect the underlying problems with energy production in the brain and other affected tissues [29].

MRI is the imaging modality of choice for evaluating CNS changes in patients with primary mitochondrial disorders(PMDs). There are no pathognomonic MRI findings associated with PMDs. Conventional MRI features are variable, ranging from highly specific to nonspecific findings and normal imaging results, particularly in cases of pure mitochondrial myopathy [29].

Common CNS PMDs with characteristic imaging phenotypes include Leigh syndrome, POLG-related disorders (POLG-RDs), Mitochondrial Encephalomyopathy, Lactic Acidosis, and Stroke-like Episodes(MELAS), Kearns-Sayre syndrome, Leber hereditary optic neuropathy (LHON), pyruvate dehydrogenase (PDH) complex deficiency, coenzyme Q10 (CoQ10) deficiency, and leukoencephalopathy with brainstem and spinal cord involvement and lactate elevation (LBSL) [24]. The neuroimaging spectrum of PMDs is broad and often complex, encompassing basal ganglia signal intensity changes, basal ganglia calcifications, cortical signal abnormalities and malformations, subependymal cysts, and various white matter alterations such as leukoencephalopathy, white matter cavitation, callosal agenesis or dysgenesis, and delayed or hypomyelination. Despite these recognizable patterns, many are nonspecific and require correlation with clinical and basic biochemical findings for accurate diagnosis, which is especially important in low-resource settings [30].

MELAS syndrome specifically presents with distinctive “stroke-like” lesions affecting the cortical and subcortical regions that characteristically do not correspond to vascular territories, unlike conventional ischemic strokes. These lesions may dynamically evolve with potential partial or complete resolution, reflecting underlying mitochondrial dysfunction rather than vascular pathology [31, 32]. Despite these patterns, many imaging findings remain nonspecific, necessitating clinical correlation for an accurate diagnosis [31, 32].

Common findings include symmetrical involvement of the thalamus, brainstem, and cerebellum, especially in syndromes like Leigh syndrome. In these cases, MRI often shows progressive and persistent lesions. Conditions such as Myoclonic Epilepsy with Ragged-Red Fibers (MERRF) often present with significant cerebellar atrophy, affecting coordination and balance [33]. Where available, magnetic resonance spectroscopy (MRS) may support the diagnosis by detecting elevated lactate, but its use is often restricted to specialized centers [34].

**Fig. 2:**
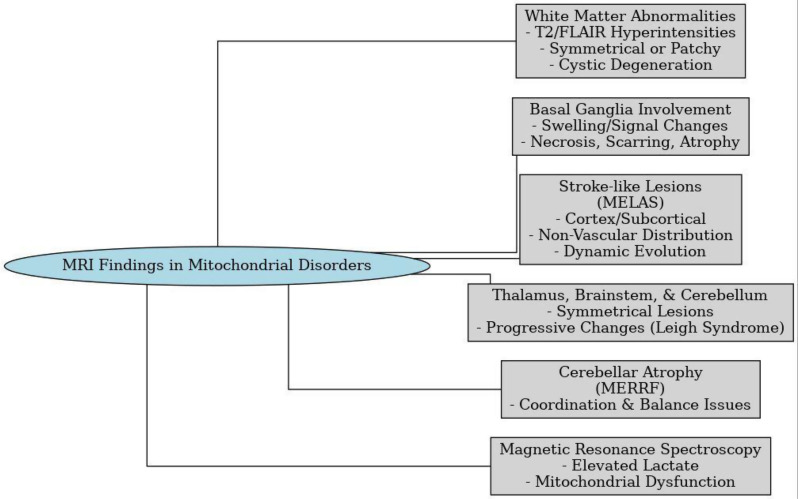
MRI Features in mitochondrial disorders

In summary, MRI findings in mitochondrial disorders vary widely but often include white matter changes, basal ganglia involvement, stroke-like lesions, and progressive atrophy. When combined with clinical history and affordable laboratory tests (e.g., serum or CSF lactate, metabolic screening), these MRI abnormalities provide a practical and accessible diagnostic pathway in most settings, reserving advanced methods such as MRS or exome sequencing for select cases [29] (Fig. 2).

### Genetic findings in mitochondrial disorder

Genetic diagnosis of mitochondrial diseases is highly challenging due to the extensive genetic heterogeneity involving over 300 nuclear genes and 37 mitochondrial DNA (mtDNA) genes. Several factors complicate the diagnostic process, including:

- Poor Genotype-Phenotype Correlations: The correlation between genetic variants and clinical symptoms can be weak or inconsistent.
- Variable Clinical Presentations: Mitochondrial diseases can manifest in a wide range of symptoms, often affecting multiple organ systems.
- Locus Heterogeneity: Multiple genes may be implicated in similar phenotypic expressions, making it difficult to pinpoint a single causal variant.
- Incomplete Penetrance: Some genetic variants may not always result in clinical disease, making it harder to diagnose based on genetic findings alone.
- Environmental Factors: Environmental influences and life stressors can exacerbate mitochondrial disorders, further complicating the diagnosis.

Moreover, the unique biological aspects of maternal inheritance of mtDNA-related pathogenic variants play a critical role in the diagnosis and interpretation of mitochondrial diseases [35].

Each complex in the electron transport chain may be encoded by either nuclear genes (nuclear-encoded) or mitochondrial DNA (mtDNA-encoded) genes. This distinction can be helpful for diagnostic strategies, as it requires the combination of WES for nuclear-encoded genes and mtDNA sequencing for mitochondrial-encoded genes to ensure accurate identification of variants [36].

Details of Electron Transport Chain Complexes and Associated Genes are summarized in Table 1.

The following Table summarizes the details of each complex in the electron transport chain, including the nuclear-encoded genes (detectable by WES) and mitochondrial-encoded genes (detectable by mitochondrial DNA sequencing) associated with each complex [7].

**Table 1:** Electron transport chain complexes and other mitochondrial genes

Complex/Function	Nuclear-Encoded Genes (Detectable by WES)	Mitochondrial-Encoded Genes (Detectable by mtDNA Sequencing)	Other Mitochondrial Functions
Complex I (NADH: Ubiquinone Oxidoreductase)	NDUFA, NDUFV	ND1, ND2, ND3, ND4, ND5, ND6	-
Complex II (Succinate Dehydrogenase)	SDHA, SDHB, SDHC, SDHD	None	-
Complex III (Cytochrome b-c1 Complex)	Cytochrome b-c1 subunits	CYTB	-
Complex IV (Cytochrome c Oxidase)	COX6A1, COX10	COX1, COX2, COX3	-
Complex V (ATP Synthase)	ATP5A, ATP5B	MT-ATP6, MT-ATP8	-
mtDNA Maintenance	POLG, TWNK	-	DNA replication and repair
Mitochondrial Dynamics	OPA1, MFN2	-	Mitochondrial fusion/fission
tRNA Synthetases	AARS2, EARS2	-	tRNA processing

### Scenario 1: when to suspect a metabolic disorder and use a scoring system

The mitochondrial disease scoring system presented in Table 2 is adapted from the Mitochondrial Disease Criteria (MDC) developed by Witters et al. [37], designed to standardize the diagnostic evaluation of mitochondrial disorders. We have tailored this system for the Iranian population, incorporating expert consensus to account for local clinical practices and resource constraints. This scoring system is particularly valuable in resource-limited settings, where access to advanced molecular testing may be restricted, as it provides a systematic framework to assess the likelihood of mitochondrial dysfunction based on clinical, metabolic, and imaging findings.” [37].

**Table 2:** Mitochondrial scoring categories

Category	Subcategory	Max Score
Clinical	Myopathy (Abnormal EMG)	2
Clinical	Motor, Neurologic Developmental Delay, or ID	2
Clinical	Multisystem (Gastrointestinal, Growth Delay, Endocrine, Immune, Vision, Renal, Cardiomyopathy)	3
Clinical	Dystonia, Ataxia, Spasticity, Neuropathy, Seizures or Encephalopathy	1
Metabolic	Plasma Lactate High ≥ 2×	2
Metabolic	Plasma Alanine High ≥ 2×	1
Metabolic	Krebs Cycle Intermediates	1
Metabolic	Organic Acids (Ethylmalonic, 3-Methylglutaconic, etc.)	1
Imaging	Leigh Disease	2
Imaging	Stroke-like Episodes	2
Imaging	Lactate Peak on MRS	1
Imaging	Leukoencephalopathy (Thalamus, Brainstem, Spinal Cord, Corpus Callosum, etc.)	1

The total score for each patient is calculated to estimate the probability of a mitochondrial disorder. Higher scores suggest a greater likelihood of mitochondrial dysfunction, enabling clinicians to prioritize diagnostic and therapeutic approaches accordingly. Based on the Mitochondrial Disease Criteria (MDC) Score, the probability of a mitochondrial disorder is classified into four categories, as summarized in Table 3 [37]. It is in noteworthy that in resource-limited settings, such as many regions in Iran, the MDC scoring system serves as a practical tool to prioritize patients for further diagnostic workup when molecular testing is not readily available.

**Table 3:** Diagnosis based on the mitochondrial disease criteria (MDC) score

MDC Score	Probability of Mitochondrial Disorder
1	Unlikely (Very Low Probability)
2–4	Possible (Moderate Probability)
5–7	Probable (High Probability)
≥ 8	Definite Mitochondrial Disorder (Confirmed)

This scoring system provides a standardized framework to assess the likelihood of mitochondrial dysfunction, ensuring a systematic approach to diagnosis and management.

### Applying the mitochondrial disease criteria (MDC) score: a practical example

For instance, consider a patient presenting with developmental delay (2 points), elevated lactate levels (2 points), and imaging findings consistent with Leigh’s disease (2 points). The total score for this patient would be 6, which falls under the “Probable” category of the scoring system. This suggests a high likelihood of a mitochondrial disorder and warrants further diagnostic investigations, such as genetic testing or functional studies.

This scoring system simplifies the diagnostic process by categorizing clinical symptoms, imaging results, and metabolic abnormalities. It ensures that even complex cases can be systematically evaluated and provides a standardized approach for clinicians to assess the probability of mitochondrial disorders.

### Senario2: approach to genetic diagnosis and management

When there is a high index of suspicion for mitochondrial disease, a detailed clinical assessment and examination should be performed. Pedigree analysis is essential to identify inheritance patterns of the disorder. The interpretation of the pedigree should take the following points into consideration:

Autosomal Dominant: Present in every generation.

Autosomal Recessive: May skip generations; carriers are typically unaffected but can pass the gene.

X-linked Recessive: More common in males, often inherited from carrier mothers.

X-linked Dominant: affects both genders but tends to be more severe in males.

Mitochondrial: Inherited exclusively from mothers to all offspring.

It is helpful to assess gender differences and identify affected individuals in each generation.

Once the initial clinical evaluation is completed, non-invasive laboratory screening should include blood and urine tests to evaluate mitochondrial dysfunction and screen for other systemic issues.

Genetic diagnostic testing should be performed, including:

1. WES, ideally performed on blood or saliva samples from both the patient and their parents.
2. Mitochondrial DNA sequencing (NGS), using blood, urine, saliva, or hair samples.

If the results are positive at this stage, treatment and management should begin. If the results are negative, additional testing should be conducted.

For negative or inconclusive results, further advanced testing options include whole genome sequencing and tissue biopsy. Biopsies can be taken from the skin, skeletal muscles, or the liver (if symptomatic) to perform histology, enzyme activity tests, and RNA analysis [35].

**Fig. 3:**
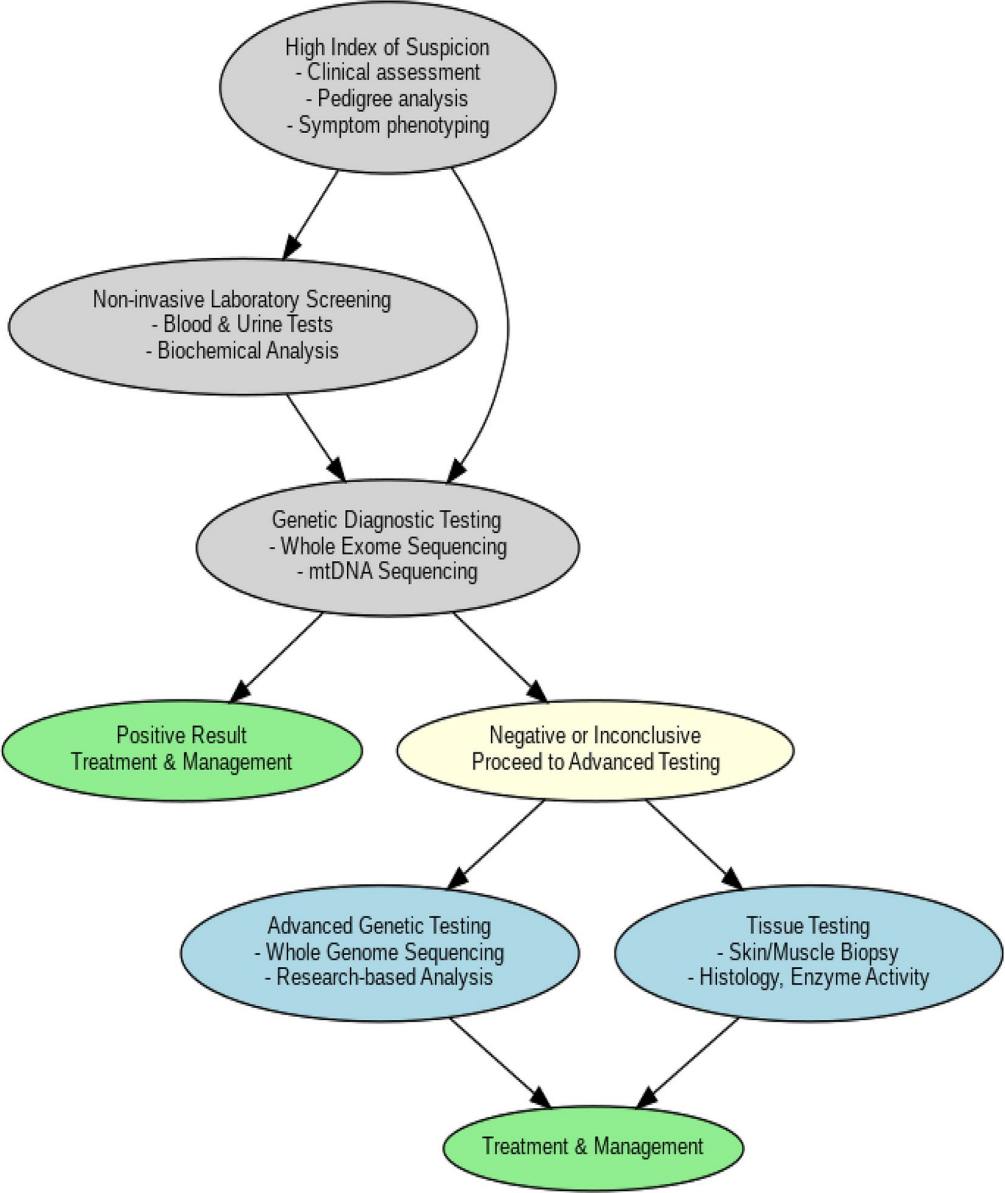
Genetic evaluation in mitochondrial disorders

If the results are positive, treatment and management should commence. If negative, other diagnostic approaches should be explored (Fig. 3).

It is noteworthy that while appreciating that access to this type of testing is variable throughout the world, many places are using genome sequencing as a first- line test, skipping whole exome sequencing. Perhaps this could be considered as an option.

### Senario3: mitochondrial disorder treatment

Mitochondrial disorders are managed through supportive treatments designed to improve energy metabolism and enhance cellular function. A key approach, known as " cocktail Therapy,” focuses on supplying essential nutrients that optimize mitochondrial performance and overall cellular health [8].

A central component of this therapy is the Vitamin B50 Complex, which contains a blend of key B vitamins. These vitamins include:

- Vitamin B1 (Thiamine).
- Vitamin B2 (Riboflavin).
- Vitamin B3 (Niacin).
- Vitamin B5 (Pantothenic Acid).
- Vitamin B6 (Pyridoxine).
- Vitamin B7 (Biotin).
- Vitamin B9 (Folic Acid).
- Vitamin B12 (Cobalamin).

These vitamins are essential for:

- Energy Production: Supporting the mitochondrial energy cycle.
- Cellular Function: Enhancing metabolic pathways critical for maintaining cell health.

Cocktail therapy offers a structured yet adaptable approach to managing mitochondrial disorders by combining multiple supplements that support cellular energy metabolism, reduce oxidative stress, and address systemic dysfunctions. As shown in Table 4, while a general regimen aims to enhance overall mitochondrial function, specialized regimens are tailored to individual clinical presentations—such as using high-dose Coenzyme Q10 for severe energy deficits or L-arginine and citrulline for managing stroke-like episodes in MELAS. In cases with elevated lactate-to-pyruvate ratios or specific genetic conditions like POLG variants, components such as biotin, folinic acid, or riboflavin are selectively emphasized. This targeted strategy underscores the importance of personalizing treatment based on symptom severity, metabolic profiles, and genetic findings. Nonetheless, given the variability in clinical practices and the limited high-quality evidence for some supplements, further research is essential to refine these regimens and establish clear, evidence-based guidelines [38].

In general, the treatment of mitochondrial disorders can be summarized in Table 4.

**Table 4:** Treatment of mitochondrial disorders

Supplement	Purpose in General Regimen	Purpose in Specialized Regimen	Targeted Pathway / Mechanism
Multivitamin	Supports overall body functions	Same as in the general regimen	Provides cofactors for multiple enzymatic reactions in energy metabolism
Vitamin B50 Complex	Enhances energy metabolism	Higher doses for increased energy demands	Supports mitochondrial enzymatic reactions including TCA cycle and electron transport chain (ETC)
Coenzyme Q10 (Ubiquinol)	Boosts cellular energy production	Higher doses for severe energy deficiencies	Electron carrier in **Complex I–III–IV** of the ETC, improves ATP synthesis
Antioxidants (Vitamin E / Alpha-lipoic acid + Biotin)	Protects against oxidative stress	For conditions like elevated lactate	Scavenges reactive oxygen species (ROS), regenerates antioxidants, supports **pyruvate dehydrogenase**
Riboflavin (B2)	Improves energy metabolism	For migraines or Complex I deficiencies	Precursor of **FAD**, cofactor for **Complex I & II** in ETC
Biotin	Supports metabolism and cellular functions	Higher doses for elevated lactate-to-pyruvate ratios	Cofactor for **carboxylases** involved in gluconeogenesis, fatty acid synthesis, and amino acid metabolism
Thiamine (B1)	Supports the nervous system and energy metabolism	For specific metabolic disorders	Cofactor for **pyruvate dehydrogenase**, α-ketoglutarate dehydrogenase, and transketolase
L-Creatine	Reduces fatigue and supports muscle health	For myopathy and exercise intolerance	Increases phosphocreatine pools for rapid ATP regeneration in mitochondria
Arginine	Supports brain function	For stroke-like episodes	Precursor of **nitric oxide**, improves vascular perfusion and mitochondrial blood flow
Folinic Acid	Supports nervous system and brain health	For refractory seizures or neuropsychiatric conditions	Supports **one-carbon metabolism**, DNA synthesis, and repair in neurons
Lutein	Protects retinal health	For pigmentary retinopathy	Antioxidant in photoreceptors, reduces oxidative damage in retinal mitochondria
Citrulline	Reduces effects of cellular stroke	For refractory stroke episodes	Enhances **arginine–nitric oxide pathway**, improves cerebral perfusion
Taurine	Protects against cellular stress	For refractory stroke episodes and other conditions	Maintains mitochondrial membrane potential, regulates calcium homeostasis
N-Acetylcysteine	Enhances the body’s antioxidant system	For plasma glutathione deficiencies	Precursor of **glutathione**, reduces oxidative stress and supports mitochondrial redox balance

The mitochondrial cocktail listed in Table 5 represents a common empiric approach used in Iran to support mitochondrial function, particularly in resource-limited settings. However, this extensive combination of supplements lacks disease-specific targeting, and the mechanisms of action or metabolic pathways influenced by each component are not always clearly delineated. Evidence supporting the efficacy of these agents varies, with stronger support for some (e.g., Coenzyme Q10, L-carnitine, riboflavin) and limited or condition-specific justification for others. For example, L-arginine and citrulline may have clearer roles in managing stroke-like episodes in MELAS, while folinic acid is considered for POLG-related disorders. Given the heterogeneity of mitochondrial diseases, a tailored, evidence-based supplementation strategy is advisable, rather than a uniform cocktail for all patients. We recommend future local protocols to prioritize components with the strongest clinical and biochemical rationale and to consider disease-specific customization based on genetic and metabolic profiles.

**Table 5:** The cocktail that is used in Iran

Supplement	Dose
Coenzyme Q10	5–15 mg/kg/day
L-carnitine	30–100 mg/kg/day
Vitamin B2 (Riboflavin)	50–200 mg/kg/day
Vitamin B1 (Thiamine)	50–100 mg/kg/day
Vitamin E	~ 4.5–9 mg/kg/day
Vitamin C	~ 3.3–16.7 mg/kg/day
Biotin	~ 0.08–0.33 mg/kg/day
Folic Acid	~ 0.03–0.33 mg/kg/day
Oral L-arginine	300–500 mg/kg/day

30 mg/kg in pediatric cases in primary co-enzyme Q10 biosynthetic defect in stroke-like episodes. Note Folinic acid should be considered in mitochondrial disease. Patients with CNS manifestations and with documented CSF deficiency or with disease states known to be associated with deficiency

### Scenario 4: management of mitochondrial disease during acute illness

Mitochondrial patients should avoid activities that lead to catabolism, including prolonged fasting. Evaluation in these cases during acute decompensations includes routine biochemical tests, liver function tests, and lactate. Other tests may be requested based on the patient’s condition. The possibility of cardiac and neurological decompensation should also be considered. Treatment of an acute attack includes glucose-containing fluids, cessation of exposure to toxic medications, and correction of any metabolic abnormalities.

#### Note

: Use of serum glucose in limited amounts is recommended in certain cases, including suspected or confirmed disorders of pyruvate metabolism, individuals on a ketogenic diet, and individuals with adverse responses to high doses of glucose.

The rate of intravenous fluids should be adjusted based on the patient’s clinical status. Outpatient mitochondrial therapies should be continued. Lipids can be used if needed even in the presence of secondary fatty acid oxidation dysfunction. These drugs should be avoided or given with caution, including corticosteroids, valproic acid, phenytoin, barbiturates, beta-blocking agents, amiodarone, nucleoside reverse transcriptase inhibitors, statins, metformin, high-dose acetaminophen, selected antibiotics including aminoglycosides, tetracycline, linezolid, azithromycin, and erythromycin. Repeat neuroimaging is recommended in any mitochondrial disease with an acute change in neurologic status [8].

### Anesthesia in mitochondrial disorders

Mitochondrial patients are at risk of complications from anesthesia. These patients are sensitive to impaired consciousness with the use of anesthetic drugs, and lactic acidosis is one of the other complications [8, 39].

In cases of general anesthesia in these patients, the following should be observed:

Minimum fasting should be considered [8, 39].

Fluids containing glucose such as dextrose saline can be used [39].

#### Note

: Fluids containing glucose are not recommended in patients on a ketogenic diet and adverse effects from giving high glucose [8, 39].

Caution is recommended in the use of volatile anesthetic drugs [8].

Caution is recommended in the use of muscle relaxants, especially in patients with muscle involvement and reduced respiratory drive [8].

These patients are prone to propofol infusion syndrome and this drug should not be used if possible or should be used only in short procedures [8, 40] Slow titration of anesthetic agents is performed to minimize hemodynamic changes in these patients. Local anesthesia is usually well tolerated. Malignant hyperthermia precautions are recommended [8, 39].

Normothermia and normoglycemia should be maintained and metabolic stress avoided [40]. Spinal or epidural anesthesia should be avoided in cases of spinal cord disorders and severe peripheral neuropathy [39].

## Conclusion

In conclusion, mitochondrial disorders are complex, multisystemic diseases resulting from impaired cellular energy production, predominantly affecting high-energy-demand organs. Accurate diagnosis requires an integrated approach combining clinical assessment, biochemical markers, neuroimaging, and genetic testing, with each modality offering critical insights. While current treatments are largely supportive, emerging therapies such as gene therapy and mitochondrial replacement hold promise for the future. In resource-limited settings, diagnostic and therapeutic challenges persist due to limited access and high costs. Therefore, the development of practical, evidence-based guidelines tailored to available resources is essential. This review aimed to address these needs by providing a comprehensive framework to support healthcare professionals in delivering effective care to affected individuals.

## Data Availability

Not applicable.
